# Cocaine-mediated circadian reprogramming in the striatum through dopamine D2R and PPARγ activation

**DOI:** 10.1038/s41467-020-18200-6

**Published:** 2020-09-07

**Authors:** Karen Brami-Cherrier, Robert G. Lewis, Marlene Cervantes, Yu Liu, Paola Tognini, Pierre Baldi, Paolo Sassone-Corsi, Emiliana Borrelli

**Affiliations:** 1 Center for Epigenetics and Metabolism, INSERM U1233, Department of Microbiology and Molecular Genetics, University of California Irvine, Irvine, CA 92697 USA; 2 Center for Epigenetics and Metabolism, INSERM U1233, Department of Biological Chemistry, University of California Irvine, Irvine, CA 92697 USA; 3 Institute for Genomics and Bioinformatics, Department of Computer Science, University of California Irvine, Irvine, CA 92697 USA

**Keywords:** Circadian rhythms, Striatum, Reward

## Abstract

Substance abuse disorders are linked to alteration of circadian rhythms, although the molecular and neuronal pathways implicated have not been fully elucidated. Addictive drugs, such as cocaine, induce a rapid increase of dopamine levels in the brain. Here, we show that acute administration of cocaine triggers reprogramming in circadian gene expression in the striatum, an area involved in psychomotor and rewarding effects of drugs. This process involves the activation of peroxisome protein activator receptor gamma (PPARγ), a nuclear receptor involved in inflammatory responses. PPARγ reprogramming is altered in mice with cell-specific ablation of the dopamine D2 receptor (D2R) in the striatal medium spiny neurons (MSNs) (iMSN-D2RKO). Administration of a specific PPARγ agonist in iMSN-D2RKO mice elicits substantial rescue of cocaine-dependent control of circadian genes. These findings have potential implications for development of strategies to treat substance abuse disorders.

## Abstract (web summary)

Drugs of abuse have been shown to perturb circadian rhythms. Here, the authors show in mice that cocaine exposure modulates circadian gene expression in the striatum through a previously unappreciated pathway that involves dopamine D2 receptors and the nuclear receptor PPARγ.

## Introduction

A large variety of fundamental biological processes, ranging from the sleep-wake cycle and metabolism, to immune responses and behavior, is regulated by the circadian clock^1,2^. In mammals, the central clock is located in the suprachiasmatic nucleus (SCN) within the hypothalamus that is entrained by light as an external *zeitgeber* (time-giver)^3^. As master regulator of organismal circadian rhythms, the SCN is thought to orchestrate the phase of oscillation of extra-SCN clocks^4^. Peripheral clocks are present in virtually all organs and cells within the body and recent findings have revealed that clocks communicate in order to achieve systemic homeostasis^5,6^. Diverse environmental cues, such as feeding behavior, also act as robust *zeitgebers* for peripheral clocks in metabolic tissues through mechanisms that appear SCN-independent^7,8^. From a molecular standpoint, the circadian clock drives oscillations in expression of a large number of genes through transcriptional-translational feedback loops composed of cycling activators and inhibitors^9^.

Drugs of abuse have been shown to induce severe perturbation of circadian rhythms^10–12^, such as disruption of the sleep/wake cycle, eating habits, blood pressure, hormone secretion and body temperature^13,14^. Importantly, desynchronization of circadian rhythms has been linked to the switch from recreational consumption to addictive behavior^15^. Cocaine, as well as other psychoactive drugs, affects the function of brain circuits such as the basal ganglia by increasing neurotransmitter release to the medium spiny neurons (MSNs), which are the principle striatal neurons and the striatum’s only output neurons. This is the case for cocaine-mediated activation of dopamine (DA) signaling in MSNs that leads to long-term adaptations of cellular programs and behavioral responses^16,17^. Importantly, there are indications that DA signaling impacts central and peripheral circadian rhythms^18,19^. In the striatum, DA levels oscillate in a circadian manner^20,21^ and are involved in the regulation of the neuronal circadian clock gene expression^22,23^. To date, however, characterization of the molecular mechanisms by which drugs of abuse alter circadian rhythms in a tissue-specific manner remains incomplete.

Under physiological conditions, the endogenous clocks coordinate transcriptional and metabolic cycles in distinct organs^7,8^. The capacity of peripheral clocks to be highly flexible through transcriptional and metabolic reprogramming is highlighted by experiments involving nutritional challenges such as fasting, high fat diet, ketogenic diet or caloric restriction^24–26^. It is unclear whether neuronal clocks are capable of similar reprogramming. We hypothesized that the short and long-term adaptation of neuronal circuits in response to cocaine would involve changes in circadian rhythmicity within the ventral striatum and in particular the Nucleus Accumbens (NAcc).

Our recent findings show that D2R-mediated signaling in MSNs critically modulates striatal responses to cocaine^27,28^. These results indicate that D2R signaling plays a critical role in the mechanisms by which drugs of abuse affect striatal physiological responses. Thus, we explored how the circadian program of striatal neurons is influenced by acute administration of cocaine in WT mice and in mutants with D2R ablation exclusively in D2R-expressing MSNs (iMSN-D2RKO mice)^27,29^. Our results show that cocaine induces a drastic reprogramming of the diurnal transcriptome in the NAcc. There is a remarkable difference in the number, type and cycling profiles of cocaine-driven oscillatory genes in iMSN-D2RKO mice. Using combined metabolomic and transcriptomic approaches, we show that D2R in iMSNs contributes to cocaine-induced activation of peroxisome protein activator receptor gamma (PPARγ), a nuclear receptor implicated in inflammatory responses^30,31^. PPARγ drives a significant fraction of de novo cocaine-induced transcriptional response, which is impaired in the absence of D2R signaling in iMSNs. Pharmacological activation of PPARγ by pioglitazone^32^ in iMSN-D2RKO mice leads to restoration of the cocaine-induced profile of circadian gene expression. Our findings unveil a D2R signaling-PPARγ connection in circadian regulation linked to cocaine-mediated rewiring in striatal neurons.

## Results

### Response of core clock genes to cocaine in both WT and iMSN-D2RKO mice

**Fig. 1:**
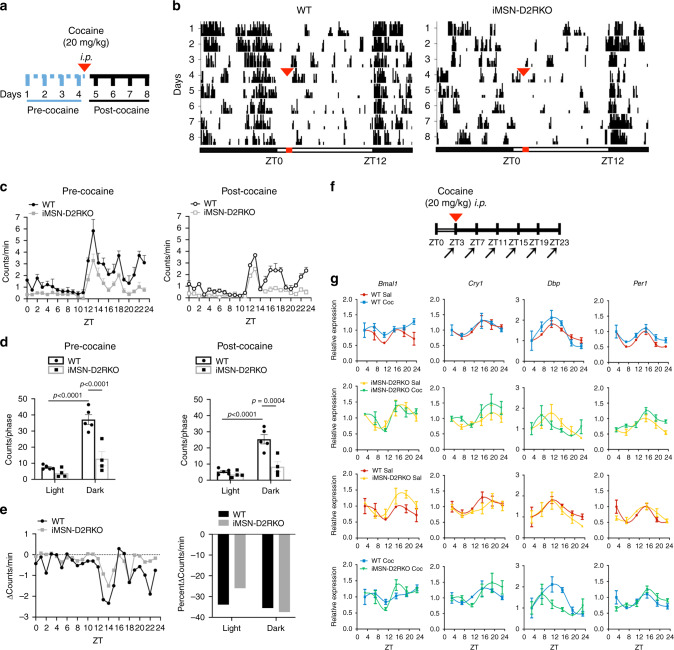
Effects of acute cocaine on circadian clock genes. **a** Schematic of the experimental design in **b**. **b** Actograms of 24 h locomotor activity in 12h-Light/12h-Dark cycles in WT (*n* = 5) and iMSN-D2RKO mice (*n* = 4) during the 4 days preceding and following cocaine administration. Cocaine (20 mg kg^-1^, i.p.) was given once at ZT3 on day 4 (red arrowheads in 1a and 1b). **c** Locomotor activity analyses from b in WT (circles) and iMSN-D2RKO (squares) mice. Graphical representation of the number of beam breaks/min/circadian time (ZT) during the days preceding (Pre-Cocaine; left) or following cocaine injection (Post-Cocaine; right). **d** Total beam breaks per phase in WT (circles) (*n* = 5) and iMSN-D2RKO (squares) (*n* = 4); inactive phase (Light), active phase (Dark), Pre-cocaine (two-way ANOVA, Genotype: F_(1, 14)_ = 27.31, *p* = 0.0001; Time: F_(1, 14)_ = 52.41, *p* < 0.0001; Interaction: F_(1, 14)_ = 15.20, *p* = 0.0016) and Post-cocaine (two-way ANOVA, Genotype: F_(1, 14)_ = 18.15, *p* = 0.0008; Time: F_(1, 14)_ = 31.86, *p* < 0.0001; Interaction: F_(1, 14)_ = 12.01, *p* = 0.0038). Tukey’s multiple comparison test *p*-values as indicated. **e** Left: The mean change in counts/min at each time point in pre-cocaine vs post-cocaine time in WT (circles) and iMSN-D2RKO (squares). Right: same as e) *left*, but represented as percent change during the light (ZT0-11) and dark (ZT12-23) phases. **f** Schematic of the circadian experimental design in g. Mice were injected with cocaine (20 mg kg^−1^, i.p) at ZT3 and NAcc samples were collected every 4 h following cocaine injection at ZT 3, 7, 11, 15, 19 and 23 (arrows). **g** Expression of core clock and clock-controlled genes: *Bmal1, Cry1, Dbp*, and *Per1* in WT saline (Sal; red circles) or cocaine (Coc; blue squares) and iMSN-D2RKO (Sal; yellow upward triangles) or (Coc; green downward triangles) analyzed by quantitative real-time PCR (*n* = 3/genotype) (three-way ANOVA*;* For statistics see Supplementary Table 1). Data are presented as mean values ± SEM.

We previously reported that iMSN-D2RKO mice display reduced motor activity in basal conditions^29^ and absence of cocaine-induced hyperlocomotion^27^. We sought to study the effects of cocaine in WT and iMSN-D2RKO mice by analyzing circadian motor activity along the daily cycle, four days before and four days after acute cocaine administration (Fig. 1a, b). Circadian motor activity was quantified as infrared beam breaks per minute at each circadian time in mice housed in home cages. Interestingly, acute cocaine does not affect the diurnal pattern of locomotor activity either in WT or iMSN-D2RKO mice, indicating that D2R deletion in iMSNs does not alter the physiology and function of the SCN central clock (Fig. 1c). Nevertheless, circadian motor activity of iMSN-D2RKO mice was decreased with respect to WT mice in the active phase before (*p* < 0.0001) and after (*p* = 0.0004) cocaine administration (Fig. 1d, e), consistent with results obtained in non-circadian behavioral settings^27,29,33^. To determine the effect of cocaine challenge on the striatal expression of clock genes, WT and iMSN-D2RKO mice received an intraperitoneal (i.p.) injection of either cocaine (Coc 20 mg kg^−1^) or saline (Sal), shortly after the beginning of the resting phase (zeitgeber time, ZT3). Animals from both groups were sacrificed every 4 h (*n* = 5 or 6/time point) and tissue punches from the NAcc collected at six time points to cover the full circadian cycle (Fig. 1f). To investigate the direct effect of cocaine in the NAcc of WT and iMSN-D2RKO mice on the core-clock machinery, we analyzed the expression of *Bmal1, Cry1, Dbp* and *Per1* in saline and cocaine-treated mice (Fig. 1g). We observed a significant effect of time for all tested core-clock genes (*p* ≤ 0.0005), indicating that expression of all genes follows the typical circadian cycle in both genotypes. There was no substantial alteration in the expression profiles of these clock genes upon cocaine treatment (*Bmal1*: *p* = 0.7389; *Cry1*: *p* = 0.7529; *Per1*: *p* = 0.6765), as well as a clock output gene, as exemplified by *Dbp* expression profile (*p* = 0.1495) (see Supplementary Table 1). A slight but nevertheless significant difference was found in *Per1* expression level between WT and iMSND2RKO mice (*p* = 0.0022). Thus, acute cocaine treatment, whether in the presence or absence of D2R in iMSNs, does not alter the rhythmic expression of this group of circadian genes.

### Reprogramming of the striatal circadian transcriptome by cocaine

Cocaine intake induces an increase of DA accumulation in the synaptic cleft through blockade of the DA transporter prolonging activation of postsynaptic neurons^34,35^. The dopaminergic mesolimbic pathway connecting the ventral tegmental area to the NAcc and cortex is critically involved in the effects of drugs of abuse^36^.

**Fig. 2:**
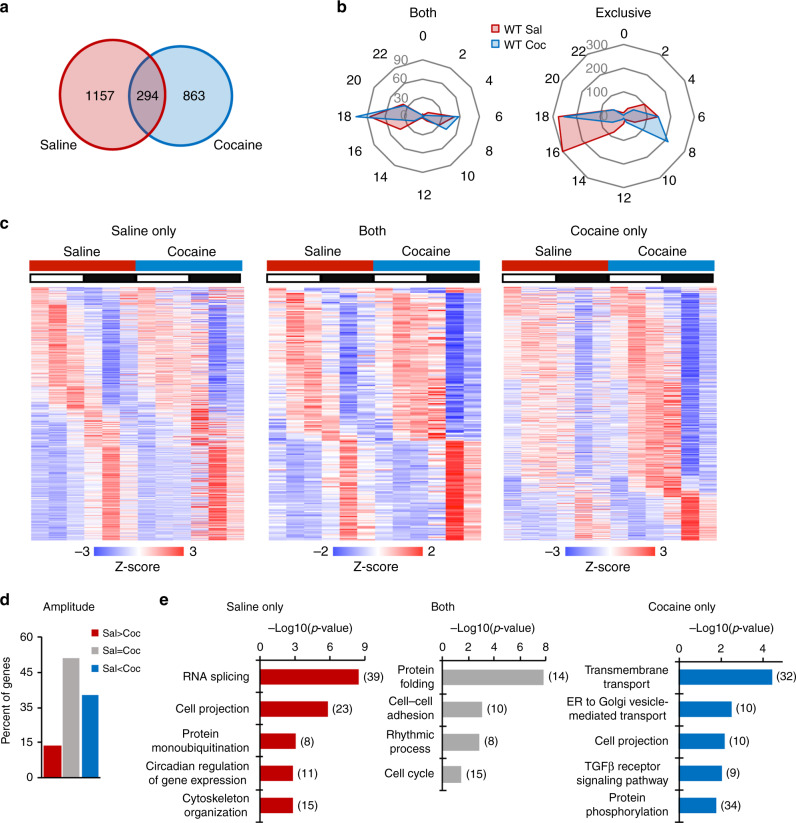
Cocaine rewires the striatal circadian transcriptome in WT mice. **a** Venn diagram representing the striatal rhythmic genes in saline and cocaine treated WT mice (*n* = 3, JTK_Cycle, cutoff *p* < 0.01). **b** Radar plots representing the phase analysis of genes whose expression is circadian in both saline (Sal) and cocaine (Coc) treated mice (left) and genes exclusively circadian in saline or cocaine conditions (right). **c**, Heat maps representing genes significantly circadian (*n* = 3, JTK_cycle, cutoff *p* < 0.01) in saline- (left), in cocaine-treated mice (right) and commonly circadian in saline and cocaine treated mice (middle). White and black bars indicate the light (ZT3, 7, 11) and dark (ZT15, 19, 23) timepoints respectively. **d** Amplitude analysis of striatal transcripts rhythmic in both saline (Sal) and cocaine (Coc) injected mice. The percentage of genes with amplitude higher, lower or equal to saline condition is reported. **e** DAVID Gene Ontology Biological Process analysis of circadian genes oscillating in saline only (left), both (middle) and cocaine only (right). Bar charts represent the -Log10(*p*-value) of each enriched term. The number of genes identified in each pathway is shown in parenthesis.

To analyze the acute cocaine-dependent genome-wide rhythmicity, RNAs were extracted from NAcc punches from brains harvested every four hours throughout a full circadian cycle (Fig. 1f) and processed for RNA-seq analyses. Rhythmic transcripts were identified using the non-parametric test JTK_CYCLE^37^, an algorithm that includes Bonferroni-adjusted multiple comparisons and incorporates a window of 20-28 h for the determination of diurnal periodicity. Out of a combined 2314 cycling transcripts identified, 1157 (50%) were rhythmic only in the saline condition. An additional 294 (~13%) were cycling in both saline- and cocaine-treated mice and, notably, 863 (~37%) de novo oscillating transcripts were identified upon cocaine challenge (Fig. 2a). The phases of oscillation of genes diurnal in both conditions were similar (Fig. 2b, c). The newly cocaine-induced oscillating transcripts display a peak at around ZT7, which is absent in saline condition. Moreover, 13% of the common oscillating genes showed a decrease in amplitude, while 35% displayed an increase upon cocaine treatment with respect to saline conditions (Fig. 2d). Thus, the circadian program of cycling genes in the NAcc is profoundly modified upon acute cocaine administration.

Pathway analyses were performed using Database for Annotation, Visualization and Integrated Discovery (DAVID) (Fig. 2e) and Reactome (Supplementary Fig. 1a–c). Both approaches identified analogous pathways in the common rhythmic transcripts between saline- and cocaine-treated NAcc. Gene Ontology (GO) annotation revealed clusters in the protein folding and rhythmic process pathways in both treatments. The saline-only specific circadian transcripts were enriched in RNA splicing, cell projection, and protein monoubiquitination pathways. Conversely, the cocaine-only specific transcripts were highly enriched in the transmembrane transport, ER to Golgi vesicle mediated transport and cell projection pathways (see Fig. 2e and Supplementary Fig. 1a, b). These results highlight the effect of a single, acute cocaine challenge on circadian function in the NAcc.

### Cocaine-driven circadian reprogramming is dependent on D2 receptors

**Fig. 3:**
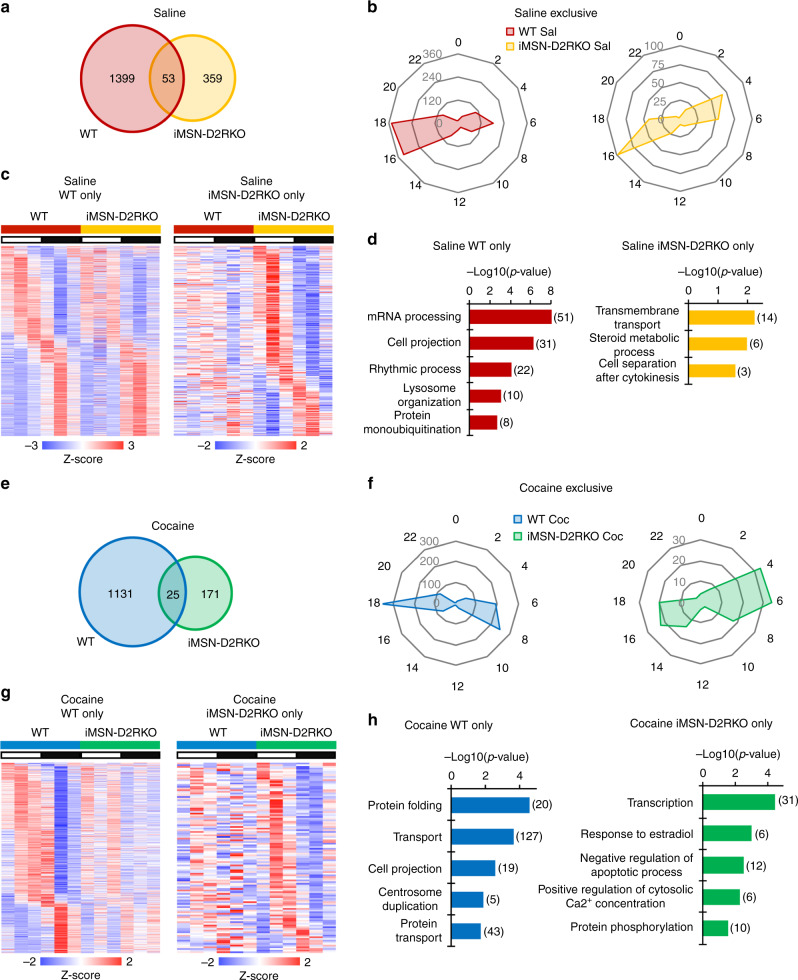
D2R ablation from iMSN reorganizes the striatal circadian transcriptome. **a** Venn diagram of striatal oscillating genes in saline treated WT and iMSN-D2RKO mice (*n* = 3, JTK_Cycle, cutoff *p* < 0.01). **b** Radar plots displaying the phase analysis of genes whose expression is exclusively circadian in WT mice (left) or in iMSN-D2RKO saline-treated (Sal) mice (right). **c** Heat maps of genes significantly circadian (*n* = 3, JTK_Cycle, cutoff *p* < 0.01) only in WT (left) or in iMSN-D2RKO (right) saline-treated mice. White and black bars indicate the light (ZT3, 7, 11) and dark (ZT15, 19, 23) timepoints respectively. **d** DAVID Gene Ontology Biological Process analysis of circadian genes oscillating in saline WT only (left) and in saline iMSN-D2RKO only (right). Bar charts represent the -Log10(*p*-value) of each enriched term. The number of genes identified in each pathway is shown in parenthesis. **e** Venn diagram of striatal oscillating genes in cocaine treated WT and iMSN-D2RKO mice (*n* = 3, JTK_Cycle, cutoff *p* < 0.01). **f** Radar plots displaying the phase analysis of genes whose expression is exclusively circadian in WT (left) or in iMSN-D2RKO cocaine-treated (Coc) mice (right). **g** Heat maps of genes significantly circadian (*n* = 3, JTK_Cycle, cutoff *p* < 0.01) only in WT (left) or in iMSN-D2RKO (right) cocaine-treated mice. White and black bars indicate the light (ZT3, 7, 11) and dark (ZT15, 19, 23) timepoints respectively. **h** DAVID Gene Ontology Biological Process analysis of circadian genes oscillating in cocaine WT only or in cocaine iMSN-D2RKO only. Bar charts represent the -Log10(*p*-value) of each enriched term. The number of genes identified in each pathway is shown in parenthesis.

Both D1R- and D2R-mediated signaling play a fundamental role in the psychomotor and rewarding properties of cocaine^38^. Importantly, mice carrying selective ablation of D2R in the iMSNs display impaired cellular and motor response to acute cocaine administration^27^. To determine the role of D2R-mediated signaling in the regulation of circadian gene oscillation in the NAcc, iMSN-D2RKO mice were treated with saline or cocaine (as shown in Fig. 1f), as previously described for their WT counterparts. RNA-seq analyses along the circadian cycle showed a substantial difference in the number of oscillatory genes as compared to WT mice in the saline condition (Fig. 3a). Indeed, we observed a drastic decrease in the number of genes oscillating in iMSN-D2RKO mice (359 transcripts) as compared to the same condition in WT mice (1399 transcripts); 53 oscillating genes were common to both genotypes (Fig. 3a). The phase of the overlapping genes was similar in both genotypes (Supplementary Fig. 2a, b) with a higher percentage of genes with greater amplitude in iMSN-D2RKO mice (Supplementary Fig. 2c). Moreover, transcripts exclusively diurnal in iMSN-D2RKO had phase distributions at approximately ZT4 and ZT16 (Fig. 3b, c). Genes oscillating only in iMSN-D2RKO mice clustered in GO annotations including transmembrane transport and steroid metabolic process (Fig. 3d and Supplementary Fig. 2d, e). It is relevant that annotation analyses of genes oscillating in both WT and iMSN-D2RKO mice include classic terms required for normal neuronal function (Supplementary Fig. 2f, g).

Acute cocaine treatment revealed a unique circadian signature in the NAcc of iMSN-D2RKO mice. Notably, a total of 1131 cycling genes were found in the cocaine-treated WT. Of these, 25 were common to iMSN-D2RKO mice. An additional 171 genes were rhythmic in iMSN-D2RKO mice only (Fig. 3e). Phase distribution analyses also revealed unique features of cocaine-induced reprogramming of circadian gene expression in WT and iMSN-D2RKO mice. Notably, a unique phase distribution peak observed at ~ZT18 in WT mice was almost completely absent in iMSN-D2RKO mice (Fig. 3f, g). On the other hand, genes oscillating in both conditions display a peak at ZT6-ZT8 (Supplementary Fig. 3a, b) and maintain similar amplitudes (Supplementary Fig. 3c). GO term analyses revealed unique pathways enriched in WT vs iMSN-D2RKO mice (Fig. 3h and Supplementary Fig. 3d, e), such as protein folding, transport, cell projection in WT, and transcription, negative regulation of apoptotic process, positive regulation of cytosolic Ca^2+^ concentration in iMSN-D2RKO. Moreover, GO annotation analyses showed a common enrichment of genes that belong to the circadian regulation of gene expression in both genotypes (Supplementary Fig. 3f, g). Taken together our results demonstrate that D2R signaling in iMSNs is critical for basal and cocaine-driven circadian oscillations in the NAcc.

### Cocaine-induced circadian response of PPARγ-target genes

**Fig. 4:**
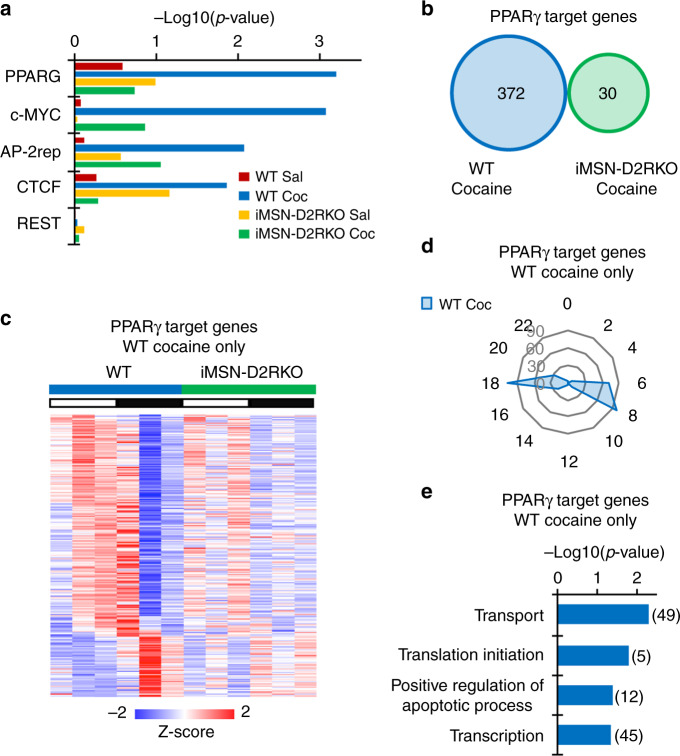
Cocaine-driven de novo oscillation of PPARγ target genes. **a** Comparison of transcription factor binding site (TFBS) analysis between WT saline (Sal), WT cocaine (Coc), iMSN-D2RKO saline, and iMSN-D2RKO cocaine. The charts report the -Log10(*p*-value). **b** Venn diagram representing the rhythmic PPARγ target transcripts after cocaine treatment in WT and iIMSN-D2RKO mice (*n* = 3, JTK_Cycle, cutoff *p* < 0.01). **c** Heat map displaying PPARγ target genes oscillating only in WT cocaine-treated mice compared to iMSN-D2RKO cocaine-treated mice (*n* = 3, JTK_Cycle, cutoff *p* < 0.01). White and black bars indicate the light (ZT3, 7, 11) and dark (ZT15, 19, 23) timepoints respectively. **d** Radar plots displaying the phase analysis of PPARγ target genes whose expression is exclusively circadian in WT cocaine-treated (Coc) mice. **e** DAVID Gene Ontology Biological Process analysis of oscillatory PPARγ target genes in cocaine-treated WT mice (*n* = 3, JTK_Cycle, cutoff *p* < 0.01). Bar charts represent the -Log10(p-value) of each enriched term. The number of genes identified in each pathway is shown in parenthesis.

To explore the molecular mechanisms by which cocaine induces de novo oscillations of striatal genes, we used MotifMap^39^ to identify transcription factor binding motifs selectively represented in rhythmic genes under saline conditions and after cocaine challenge. A profound reorganization in transcription factor pathway usage was observed upon cocaine challenge, with a significant enrichment of genes containing PPARγ binding sites for genes oscillating in WT mice (Fig. 4a). Indeed, 372 out of 863 cocaine-induced newly oscillating genes are PPARγ targets (Figs. 2a and 4b, c). Importantly, cocaine-induced enrichment of PPARγ binding sites is not observed in the iMSN-D2RKO cocaine-treated mice. Thus, ablation of D2R from iMSNs significantly reduces the cocaine-induced PPARγ oscillatory program observed in WT mice (Fig. 4b). Among the cocaine-induced PPARγ target genes, none were common oscillators in both genotypes. The significant fraction of de novo cycling PPARγ target genes induced by the first exposure to cocaine in WT mice, prompted us to further explore the involvement of this nuclear factor. Phase oscillation analyses of PPARγ cycling targets revealed a specific phase distribution in cocaine treated WT mice at ZT6-ZT8 and ZT18 (Fig. 4d). GO biological process analyses of the PPARγ-target genes in WT cocaine-treated mice revealed transport, translation initiation, positive regulation of apoptotic process, and transcription as key annotations (Fig. 4e and Supplementary Fig. 4a). Taken together, our data underscore the involvement of PPARγ signaling pathway in cocaine-induced transcriptional reprogramming of the NAcc clock. This unique cocaine-induced transcriptional feature is absent in mice with ablation of D2R from iMSNs.

### D2R-driven PPARγ nuclear enrichment upon cocaine

**Fig. 5:**
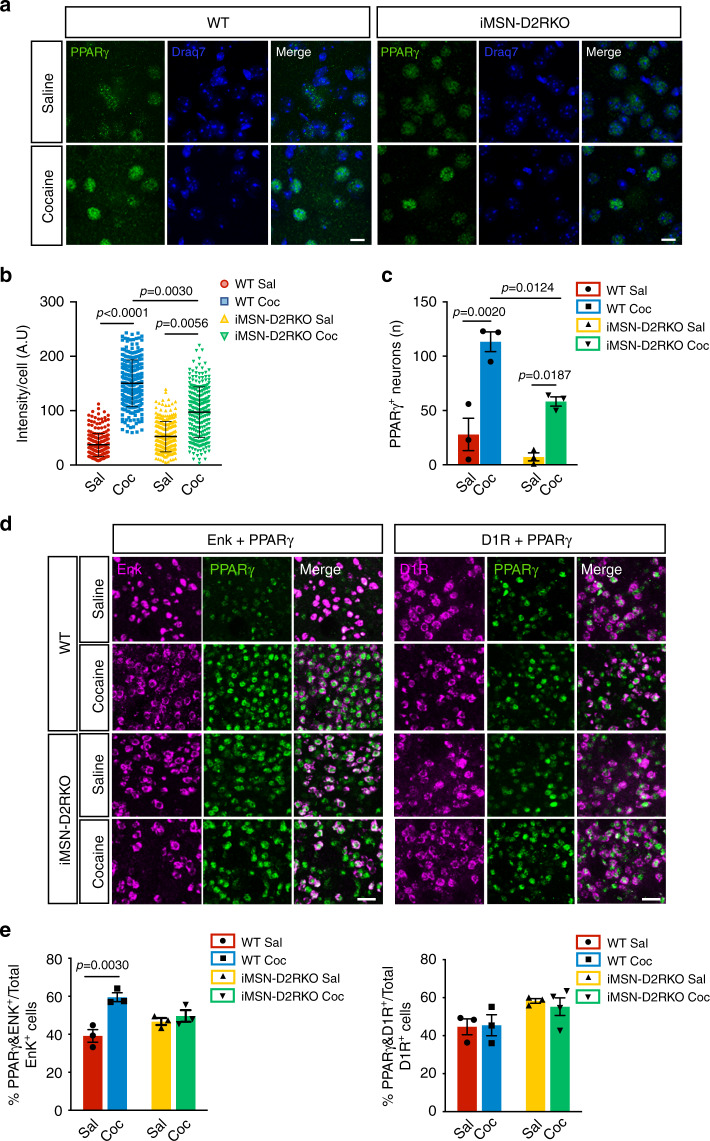
Cocaine-induced nuclear PPARγ is impaired in iMSN-D2RKO mice. **a** Immunolabeling of PPARγ and nuclei on striatal sections of saline and cocaine treated WT and iMSN-D2RKO mice. Scale bar: 25 µm. **b** Quantification of the fluorescent intensity of PPARγ immunolabeling in WT and iMSN-D2RKO mice treated with saline (Sal; WT: red circles; iMSN-D2RKO: yellow upward triangles) or cocaine (Coc; WT: blue squares; iMSN-D2RKO: green downward triangles). Data were analyzed by two-way ANOVA using the mean ± SD of intensity/cell for each biological replicate (*n* = 4/group) (Genotype: F_(1, 12)_ = 8.458, *p* = 0.0131; Treatment: F_(1, 12)_ = 91.23, *p* < 0.0001; Interaction: F_(1, 12)_ = 12.81, *p* = 0.0038), Tukey’s multiple comparison test. **c** Quantification of PPARγ positive neurons with the indicated treatment (*n* = 3/group). Significance was calculated using two-way ANOVA (Genotype: F_(1, 8)_ = 16.87, *p* = 0.0034; Treatment: F_(1, 8)_ = 54.77, *p* < 0.0001; Interaction: F_(1, 8)_ = 3.474, *p* = 0.0993) Tukey’s multiple comparison test. **d** Double in-situ/immunofluorescence for *Enkephalin* (Enk) or *D1R* mRNA and PPARγ protein in cocaine treated WT and iMSN-D2RKO mice. Scale bar: 50 µm. **e** Percentage of PPARγ- and Enk- or D1R-positive cells in cocaine and saline treated WT and iMSN-D2RKO mice (Enk: *n* = 3/group; D1R: *n* = 3 WT Sal, *n* = 3 WT Coc, *n* = 3 iMSN-D2RKO Sal, *n* = 4 iMSN-D2RKO Coc). Two-way ANOVA (Enk: Genotype: F_(1, 8)_ = 0.1782, *p* = 0.6841; Treatment: F_(1, 8)_ = 18.69, *p* = 0.0025; Interaction: F_(1, 8)_ = 10.53, *p* = 0.0118; D1R: Genotype: F_(1, 9)_ = 7.117, *p* = 0.0257; Treatment: F_(1, 9)_ = 0.06215, *p* = 0.8087; Interaction: F_(1, 9)_ = 0.1943, *p* = 0.6697). Tukey’s multiple comparison test. Data in c and e are presented as mean values ± SEM.

PPARγ localizes in the cytoplasm and upon activation translocates to the nucleus to activate transcription of specific genes^40^. We performed immunofluorescence analyses using a nuclear-specific PPARγ antibody to quantify the induction of nuclear PPARγ staining in the NAcc of WT and iMSN-D2RKO mice upon cocaine treatment. For this purpose, mice of both genotypes were administered either saline or cocaine (20 mg kg^−1^) at ZT3 and sacrificed at ZT7. A diffuse nuclear PPARγ staining was observed in NAcc neurons in saline conditions in both genotypes (Fig. 5a). After cocaine we observed a significant increase in nuclear PPARγ staining in WT NAcc neurons, which was absent in iMSN-D2RKO mice. Quantification of PPARγ nuclear intensity per cell, as well as of the number of cells with nuclear PPARγ staining, shows a statistically significant increase of nuclear PPARγ localization upon cocaine in WT as compared to iMSN-D2RKO NAcc neurons (Fig. 5b, *p* = 0.0030; Fig. 5c, *p* = 0.0124). To establish the identity of the MSNs showing the heightened intensity of PPARγ nuclear staining, we performed in situ hybridization coupled to immunohistochemistry using probes specific for the two subtypes of MSNs. This allowed for the unambiguous identification of D2R-expressing iMSNs from D1R-expressing dMSNs. Double *in-situ* hybridization/immunohistochemistry analyses were performed using riboprobes for *enkephalin* (Enk)^41^, an iMSNs specific marker or the dopamine D1 receptor (*D1R)*, a dMSN specific marker, together with the specific PPARγ antibody. These experiments demonstrated that the cocaine-driven increase in PPARγ nuclear staining occurs in iMSNs (*p* = 0.0030) and not in dMSNs (*p* = 0.9991) (Fig. 5d, e). Importantly, the increase of PPARγ in iMSNs nuclei after cocaine was not observed in the NAcc of iMSN-D2RKO mice (*p* = 0.8693) (Fig. 5d, e). These results point to a cocaine-mediated D2R-dependent activation of PPARγ.

### Lack of PPARγ activation and function in iMSN-D2RKO mice

**Fig. 6:**
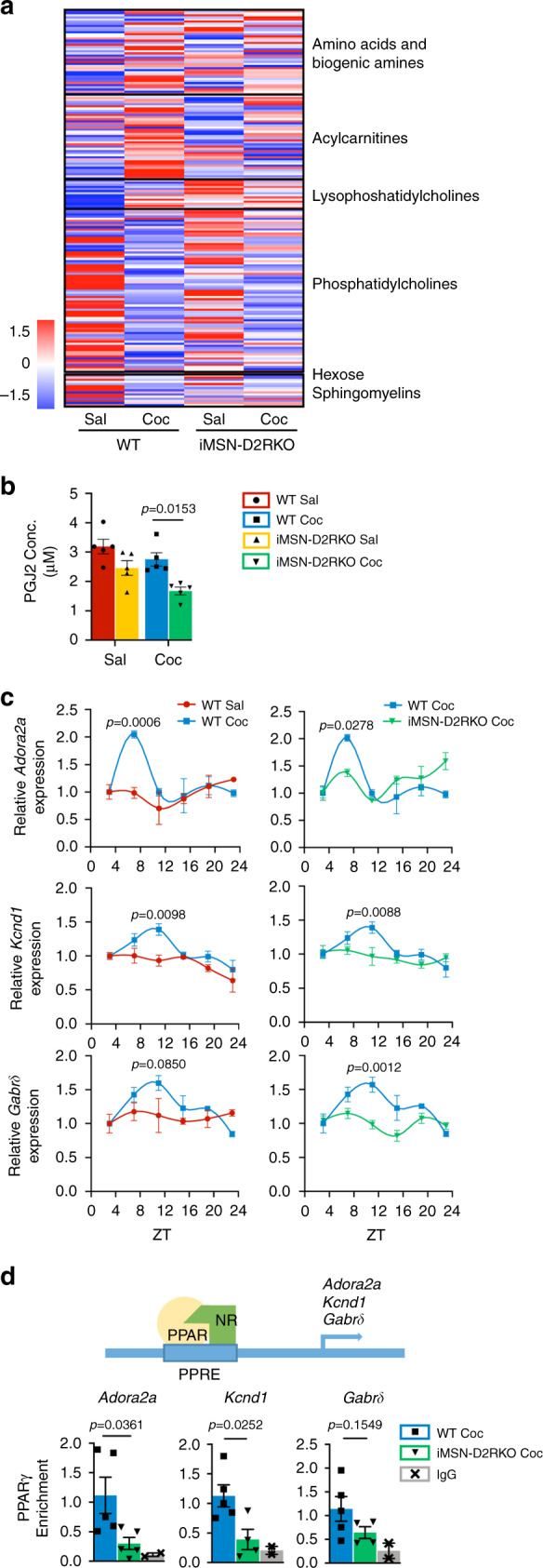
PPARγ signaling is altered in iMSN-D2RKO mice. **a** Heatmap of the 180 metabolites analyzed in WT and iMSN-D2RKO mice, either saline or cocaine-treated (*n* = 5/group). Metabolites were measured at ZT7 after saline or cocaine was injected at ZT3. Classes of metabolites measured are indicated on the right. **b** Prostaglandin PGJ2-type (15-deoxy-Δ^12,14^-PGJ2) concentration assessed by enzyme-linked immunosorbent assay (ELISA) at ZT7 in WT and iMSN-D2RKO mice treated with saline (Sal: WT, red circles; iMSN-D2RKO, yellow upward triangles) or cocaine (Coc: WT, blue squares; iMSN-D2RKO, green downward triangles) (*n* = 5/group/genotype). Two-way ANOVA (Genotype: F_(1, 16)_ = 0.1696, *p* = 0.0008; Treatment: F_(1, 16)_ = 7.719, p = 0.0134; Interaction: F_(1, 16)_ = 0.6034, *p* = 0.4486). Tukey’s multiple comparison test *p*-values as indicated. **c** Circadian expression of selected PPARγ target genes *Adora2a*, *Kcnd1*, *Gabrδ* as determined by quantitative real time PCR (*n* = 3/group). WT Sal (red circles), WT Coc (blue squares), iMSN-D2RKO Coc (green downward triangles). Two-way ANOVA *Adora2a* WT Sal vs WT Coc: Treatment: F_(1, 24)_ = 4.372, *p* = 0.0473; Time: F_(5, 24)_ = 4.253, *p* = 0.0065; Interaction: F_(5, 24)_ = 3.983, *p* = 0.0090; *Adora2a* WT Coc vs iMSN-D2RKO Coc: Genotype: F_(1, 24)_ = 0.4260, *p* = 0.5201; Time: F_(5, 24)_ = 7.429, *p* = 0.0002; Interaction: F_(5, 24)_ = 4.363, *p* = 0.0058; *Kcnd1* WT Sal vs WT Coc: Treatment: F_(1, 24)_ = 10.98, *p* = 0.0029; Time: F_(5, 24)_ = 6.118, *p* = 0.0009; Interaction: F_(5, 24)_ = 1.640, *p* = 0.1878; *Kcnd1* WT Coc vs iMSN-D2RKO Coc: Genotype: F_(1, 24)_ = 5.302, *p* = 0.0303; Time: F_(5, 24)_ = 4.360, *p* = 0.0058; Interaction: F_(5, 24)_ = 2.743, *p* = 0.0426; *Gabrδ* WT Sal vs WT Coc: Treatment: F_(1, 24)_ = 2.940, *p* = 0.0993; Time: F_(5, 24)_ = 2.690, *p* = 0.0456; Interaction: F_(5, 24)_ = 2.124, *p* = 0.0972; *Gabrδ* WT Coc vs iMSN-D2RKO Coc: Genotype: F_(1, 24)_ = 15.59, *p* = 0.0006; Time: F_(5, 24)_ = 5.308, *p* = 0.0020; Interaction: F_(5, 24)_ = 3.961, *p* = 0.0092. Bonferroni’s multiple comparison test p-values as indicated. **d**, Chromatin recruitment of PPARγ at PPAR response element (PPRE) binding site contained in *Adora2a* (*p* = 0.0361; WT Coc (blue squares) *n* = 5, iMSN-D2RKO Coc (green downward triangles) *n* = 5, IgG (gray X’s) *n* = 2), *Kcnd1* (*p* = 0.0252; WT Coc *n* = 5, *n* = 4 iMSN-D2RKO Coc, IgG *n* = 2) and *Gabrδ* promoters (*p* = 0.1549; WT Coc *n* = 5, iMSN-D2RKO Coc *n* = 4, IgG *n* = 2. unpaired Student’s t-test. Data are presented as mean values ± SEM.

To ascertain whether the metabolic consequences of acute cocaine treatment may be linked to PPARγ activation, we performed mass-spectrometry (MS) metabolomics analyses from isolated NAcc at ZT7 after either saline or cocaine (20 mg kg^−1^; i.p.) administration at ZT3. We identified a significant effect of cocaine on lipid metabolism (Fig. 6a). Among 180 metabolites analyzed, 145 were lipids including: phospholipids, acylcarnitines and sphingolipids. In WT, but not in iMSN-D2RKO mice, phosphatidylcholine levels were significantly decreased after cocaine treatment while most lysophosphatidylcholines increased. D2R activation is involved in the conversion of phosphatidylcholine into lysophosphatidylcholine and arachidonic acid (AA)^42,43^, the latter being a precursor of prostaglandins^44,45^. Importantly, prostaglandins are well-characterized PPARγ natural ligands^46^. Interestingly, AA release in the striatum is regulated by D1R and D2R in an opposite manner^45,47^; D1R signaling inhibits while D2R signaling increases AA release. Based on these findings, we reasoned that the efficient turnover of phosphatidylcholine levels in response to cocaine would be dampened in mice with D2R ablation in iMSNs. We thereby analyzed the levels of the PGJ2-type prostaglandin (15-deoxy-Δ^12,14^-PGJ2)^48^, a prostaglandin that specifically binds and activates PPARγ^49^, in the NAcc of WT and iMSN-D2RKO mice. Indeed, we observed that in response to cocaine, there is a significantly lower level of 15-deoxy-Δ^12,14^-PGJ2 in the NAcc of iMSN-D2RKO mice as compared to saline treated mice (*p* = 0.0153) (Fig. 6b). In contrast, WT mice show no significant change in 15-deoxy-Δ12,14-PGJ2 levels after cocaine treatment, a response that mirrors results obtained in human cocaine users^50^. These findings point to D2R signaling as a key player in the cocaine-driven prostaglandin production involved in PPARγ activation.

We next assessed the downstream effects of PPARγ activation by analyzing the expression of specific genes from the list of PPARγ circadian putative targets (Fig. 4c). Among these genes, *Adora2a* (Adenosine A2a Receptor), *Kcnd1* (Potassium Voltage-Gated Channel Subfamily D Member 1), and *Gabrδ* (Gamma-Aminobutyric Acid Type A Receptor Delta Subunit) are not oscillatory under normal conditions in WT mice (Fig. 6c). However, upon cocaine treatment, their expression displayed de novo oscillatory profiles. In contrast, in cocaine treated iMSN-D2RKO mice, these genes were not cyclically expressed, their circadian expression parallels that of saline-control mice (Fig. 6c). Next, we analyzed the molecular mechanism of control at the promoter level by chromatin immunoprecipitation assays (ChIP) using NAcc nuclear extracts from both WT and iMSN-D2RKO cocaine-treated mice harvested at ZT7. Using PPARγ nuclear-specific antibodies, we show that PPARγ chromatin recruitment to *Adora2a* and *Kcnd1* promoters was significantly reduced in iMSN-D2RKO animals as compared to WT (Fig. 6d) (*Adora2a:* *p* = 0.0361 and *Kcnd1*: *p* = 0.0252). An analogous trend was observed for the *Gabrδ* promoter (*p* = 0.1549). These results support a scenario in which PPARγ activation by cocaine leads to the de novo program of D2R signaling-dependent circadian genes in the NAcc.

### Rescue of PPARγ function using the specific agonist pioglitazone

**Fig. 7:**
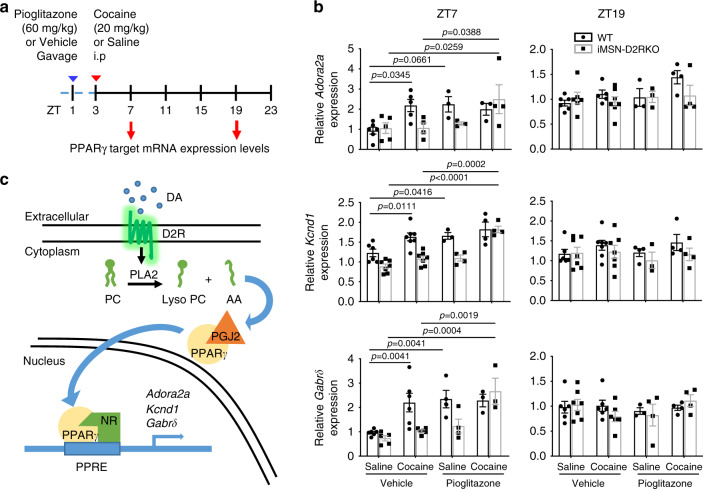
Pharmacological activation of PPARγ restores PPARγ signaling iMSN-D2RKO mice. **a** Schematic representation of the experimental design, arrowheads indicate the time and treatment of Pioglitazone (blue) and Cocaine treatments (red). **b** Expression of selected PPARγ target genes *Adora2a, Kcnd1*, and *Gabrδ* as determined by quantitative real time PCR at ZT7 and ZT19 in presence or absence of Pioglitazone (60 mg kg^−1^) prior to saline or cocaine (20 mg kg^−1^) (*Adora2a* ZT7: Genotype: F_(1, 25)_ = 2.003, *p* = 0.1693; Treatment: F_(3, 25)_ = 4.682, *p* = 0.0099; Interaction: F_(3, 25)_ = 2.479, *p* = 0.0845 (Vehicle and Saline: WT *n* = 6, iMSN-D2RKO *n* = 5; Vehicle and Cocaine: WT *n* = 5, iMSN-D2RKO *n* = 4; Pioglitazone and Saline: WT *n* = 3, iMSN-D2RKO *n* = 3; Pioglitazone and Cocaine: WT *n* = 3, iMSN-D2RKO *n* = 4); *Kcnd1* ZT7: Genotype: F_(1, 34)_ = 26.20, *p* < 0.0001; Treatment: F_(3, 34)_ = 20.05, *p* < 0.0001; Interaction: F_(3, 34)_ = 3.130, *p* = 0.0383 (Vehicle and Saline: WT *n* = 6, iMSN-D2RKO *n* = 7, Vehicle and Cocaine WT *n* = 7, iMSN-D2RKO *n* = 7; Pioglitazone and Saline: WT *n* = 3, iMSN-D2RKO *n* = 4, Pioglitazone and Cocaine WT *n* = 4, iMSN-D2RKO *n* = 4); *Gabrδ* ZT7 Genotype: F_(1, 29)_ = 7.445, *p* = 0.0107; Treatment: F_(3, 29)_ = 11.26, *p* < 0.0001; Interaction: F_(3, 29)_ = 3.378, *p* = 0.0315 (Vehicle and Saline: WT *n* = 6, iMSN-D2RKO *n* = 5, Vehicle and Cocaine: WT *n* = 6, iMSN-D2RKO *n* = 6; Pioglitazone and Saline: WT *n* = 4, iMSN-D2RKO *n* = 4; Pioglitazone and Cocaine: WT *n* = 3, iMSN-D2RKO *n* = 3); *Adora2a* ZT19: Genotype: F_(1,30)_ = 1.352, *p* = 0.2540; Treatment: F_(3, 30)_ = 2.071, *p* = 0.1250; Interaction: F_(3, 30)_ = 1.406, *p* = 0.2603 (Vehicle and Saline: WT *n* = 6, iMSN-D2RKO *n* = 6; Vehicle and Cocaine: WT *n* = 5, iMSN-D2RKO *n* = 7; Pioglitazone and Saline: WT *n* = 3, iMSN-D2RKO *n* = 3; Pioglitazone and Cocaine WT *n* = 4, iMSN-D2RKO *n* = 4); *Kcnd1* ZT19: Genotype: F_(1, 34)_ = 1.903, *p* = 0.1767; Treatment: F_(3, 34)_ = 0.7013, *p* = 0.5578; Interaction: F_(3, 34)_ = 0.3919, *p* = 0.7596 (Vehicle and Saline: WT *n* = 6, iMSN-D2RKO *n* = 7; Vehicle and Cocaine WT *n* = 7, iMSN-D2RKO *n* = 7; Pioglitazone and Saline: WT *n* = 4, iMSN-D2RKO *n* = 3; Pioglitazone and Cocaine: WT *n* = 4, iMSN-D2RKO *n* = 4); *Gabrδ* ZT19 Genotype: F_(1, 32)_ = 0.09598, *p* = 0.7587; Treatment: F_(3, 32)_ = 0.8134, *p* = 0.4959; Interaction: F_(3, 32)_ = 0.8259, *p* = 0.4893 (Vehicle and Saline: WT *n* = 6, iMSN-D2RKO *n* = 7; Vehicle and Cocaine WT *n* = 6, iMSN-D2RKO *n* = 6; Pioglitazone and Saline WT *n* = 3, iMSN-D2RKO *n* = 4; Pioglitazone and Cocaine: WT *n* = 4, iMSN-D2RKO *n* = 4)). Tukey’s multiple comparison test *p*-values as indicated. Data are presented as mean values ± SEM. **c** Simplified overview depicting D2R-mediated cocaine effect on circadian transcription of PPARγ target genes through PPARγ activation by prostaglandins (PGJ2). Dopamine (DA) activation of D2R stimulates Phospholipase A2 (PLA2) converting phosphatidylcholine (PC) to lysophosphatidylcholine (Lyso PC) and arachidonic acid (AA); AA is later converted into Prostaglandin (PGJ2). PGJ2 induces PPARδ nuclear translocation and transcriptional activation.

To validate the critical role played by PPARγ in D2R signaling-dependent circadian reprogramming upon cocaine, WT and iMSN-D2RKO mice were subjected to oral gavage with pioglitazone, a specific PPARγ activator^51^ that crosses the blood brain barrier^52^. Pioglitazone or vehicle, were administered at ZT1, 2 h before the acute cocaine injection (Fig. 7a). Expression of the *Adora2a*, *Kcnd1* and *Gabrδ* genes was analyzed at ZT7 and at ZT19 (Fig. 7b). Pioglitazone treatment before cocaine reestablished the induction of *Adora2a* (*p* = 0.0388), *Kcnd1* (*p* < 0.0001), and *Gabrδ* (*p* = 0.0019) gene expression in iMSN-D2RKO mice at ZT7, which nicely paralleled WT expression levels (Fig. 7b). Thus, PPARγ activation operates as a direct link between cocaine, D2R-signaling^42,43^ and downstream gene expression (Fig. 7c). These results identify PPARγ as a critical factor that intervenes in the transcriptional reprogramming of the striatal clock upon acute cocaine treatment.

## Discussion

Drugs of abuse, such as cocaine, are known to alter human physiology and circadian rhythms^13^. While relevant information about the molecular mechanisms by which cocaine affects short or long-term neuronal plasticity has been accumulated^53^, little is known about how it interplays with the circadian system. Deciphering how cocaine alters circadian regulation may provide critical knowledge to design strategies aimed at mitigating the daily dysfunctions of drug addicts. Previous studies have addressed this question through the analysis of chronically treated WT mice^54^ or mutant mice for specific clock genes^11,18,55,56^. In this study, we first sought to decipher how a single acute cocaine treatment affects genome-wide circadian oscillations within the NAcc, and secondly to dissect the D2R-mediated signaling pathways in striatal neurons. For this purpose, we exploited mouse models in which genetic ablation of D2R is targeted uniquely to striatal iMSNs. We demonstrate that cocaine generates a profound reprogramming of circadian gene expression and identified PPARγ as one critical player that elicits the acute effects of cocaine through D2R-mediated signaling.

D2R is essential for the psychomotor and rewarding effects of psychoactive drugs such as cocaine^27,57,58^. Indeed, constitutive D2R knockout mice self-administer higher amounts of cocaine as compared to WT littermates^58^. Importantly, lower striatal D2R levels have been observed in cocaine abusers as well as in rodent models^59–61^. Thus, ablation of D2R from the main striatal population, as achieved in iMSN-D2RKO mice, represents an ideal model to study the mechanisms by which cocaine affects striatal signaling and circuitry. Previous studies revealed the importance of D2R for intrastriatal connections (i.e. collaterals between iMSN and dMSN) necessary for the psychomotor effects of cocaine^27,29,33,62^.

Our findings place D2R in a central position in the modulation of circadian rhythmicity in the striatum. Indeed, D2R ablation in iMSNs leads to a significant reduction in the number of oscillating genes in the NAcc. Our data reinforce emerging evidence suggesting that cocaine-mediated increase of dopamine is involved in maintaining circadian rhythms in brain areas including the retina, olfactory bulb, striatum, midbrain and hypothalamus^63^. Remarkably, cocaine administration is significantly less effective on circadian reprogramming in the absence of D2R.

Previous reports have indicated that D2R-mediated signaling modulates *Clock* and *Per2* gene expression^22,23^. Our results show that D2R also modulates *Per1* expression (Supplementary Table 1). Cocaine treatment does not lead to major alterations in the circadian oscillation of the core clock genes *Bmal1, Cry1* and *Dbp* in striatal neurons. On the other hand, we observe induction of newly oscillatory genes. This is remarkable when considering the extensive changes in circadian gene expression observed between WT and iMSN-D2RKO mice. Altogether these observations point to a D2R signaling-driven cocaine-induced reprogramming of the NAcc. Our study allows the identification of PPARγ as a key mediator of cocaine-induced rhythmic transcriptional reprogramming.

While originally characterized for its role in adipogenesis and glucose metabolism, PPARγ has been recently linked to neurological disorders such as neurodegeneration and neuro-inflammation^64,65^. Importantly, we have demonstrated that D2R ablation prevents cocaine-driven PPARγ activation and the consequent de novo oscillation of PPARγ target genes. Dependence on D2R can be circumvented by the administration of the specific PPARγ agonist pioglitazone. Our results support recent findings suggesting the involvement of PPARγ in cocaine use disorder. Indeed, pioglitazone treatment during abstinence has a positive effect on cocaine addiction by reducing cocaine self-administration^66^. GO analyses of the cocaine treated WT and iMSN-D2RKO NAcc transcriptomes shows that the most significant annotation in iMSN-D2RKO is transcription factors which is absent in WT mice. This notion supports a modulatory role of D2R signaling in NAcc-dependent molecular responses to cocaine. Thus, while dMSNs have been critically involved in cocaine-mediated responses^67–72^, the modulatory role of D2R signaling needs to be further highlighted. Along these lines, it is tempting to speculate that alteration of cocaine-induced circadian reprogramming in absence of D2R might also occur in cocaine abusers where the levels of D2R are dampened^59^. Notably, the full D2R knockout mice show heightened intake of cocaine as measured in cocaine self-administration studies^58^. Since the iMSN-D2RKO mice show multiple features of the full D2R knockout mice, it is tempting to speculate that they might also self-administer higher amounts of cocaine. Future studies will address this question.

Our findings reveal the fundamental role of D2R in circadian physiology of the brain’s reward system. D2R signaling plays a crucial role in the reprogramming of diurnal transcription driven by acute cocaine in the NAcc. Unsuspected to date, D2R-mediated signaling triggers a regulatory circuit that leads to PPARγ activation. This response underlies the cyclic activation of a large number of de novo oscillatory genes. These results well exemplify the complexity underlying the effects of cocaine in the brain by adding a member of the nuclear receptor family to the molecular circuitry previously implicated in the response to cocaine^73–77^. Finally, the identification of the PPARγ pathway as a mediator of D2R signaling represents an important promising target for the clinical treatment of drug addiction.

## Methods

### Animals

iMSN-D2RKO mice were generated by mating D2R^flox/flox^ mice with D2R^flox/flox/D1R-CRE+/-^ mice^29^. In D2R^flox/flox/D1R-CRE+/-^ mice, the DA D1R promoter drives the CRE recombinase. The ability of this CRE to eliminate D2R in iMSNs^29^ resides in the common expression of D1R and D2R in embryonic MSN precursors^78^. Absence of D2R from iMSNs was previously shown by binding analyses on striatal extracts using a D2R-specific 3H-labeled ligand, as well as by double in situ hybridization experiments using GAT1 as marker of MSNs and D2R exon 2 specific probes^29^.

Mice were maintained on a standard 12 h light/ 12 h dark cycle; food and water were available ad libitum in ~25 °C and 40–60% humidity. Animals’ care and use was in accordance with guidelines of the Institutional Animal Care and Use Committee at the University of California, Irvine. Genotype identification was performed by Southern blot and PCR analyses of DNA extracted from tails biopsies.

### Drugs

Before pharmacological treatments, mice were handled for at least 3 days for 5 min. On the day of the test, mice were habituated to the novel home cage for 2 h and then administered either cocaine or saline. Cocaine (Sigma, Cat. #C5776) was dissolved in saline (NaCl 0.9%) and injected intraperitoneally (i.p.) at the dose of 20 mg kg^−1^. Pioglitazone (Cayman Chemical, Cat. # 71745) was dissolved in DMSO to have a stock solution of 10 mg mL^−1^. Pioglitazone solution was diluted 1:1 in PBS and administered 2 h prior to either cocaine or saline injection by oral gavage at a dose of 60 mg kg^−1^.

### Locomotor activity analysis

Activity was measured on individually housed mice *n* = 4–5/group for 11 days using Actimetrics optical beam motion detection (Philips Respironics). Data was collected using Minimitter Vital View v5.0 data acquisition software and analyzed through Matlab R2013a v9.7.0.1296695 software and Clocklab software v2.72.

### Quantitative RT–PCR

Striatum samples were homogenized in TRIzol lysis reagent (Thermo Fisher) following manufacturer’s instructions. Total RNA was reverse-transcribed using iScript Reverse Transcription Supermix (Biorad Cat. N. 1708840). Gene expression was analyzed by Real-Time PCR (BIO-RAD Real-Time System; BIO-RAD CFX Manager Software v3.1) using SsoAdvanced Universal SYBR Green Supermix (Biorad Cat. N. 172-5270). The sequences of the primers used for RT-PCR are Kcnd1 Forward: 5′-TCCGTTTGGCAAAGAGTGGT-3′, Kcnd1 Reverse: 5′-AGCTCGTCTGTGAACTCGTG-3′; Gabrd Forward: 5′-GGCGCCAGGGCAATGAAT-3′, Gabrd Reverse: 5′-GTCAATGCTGGCCACCTCTA-3′; Adora2a Forward: 5′-TTCATCGCCTGCTTTGTCCT-3′, Adora2a Reverse: 5′-AATGATGCCCTTCGCCTTCA-3′; Bmal1 Forward: 5′-GCAGTGCCACTGACTACCAAGA-3′, Bmal1 Reverse: 5′-TCCTGGACATTGCATTGCAT-3′; Per1 Forward: 5′-ACCAGCGTGTCATGATGACATA-3′, Per1 Reverse: 5′-GTGCACAGCACCCAGTTCCC-3′; Dbp Forward: 5′-AATGACCTTTGAACCTGATCCCGCT-3′, Dbp Reverse: 5′-GCTCCAGTACTTCTCATCCTTCTGT-3′; Cry1 Forward: 5′-CAGACTCACTCACTCAAGCAAGG-3′, Cry1 Reverse5′-TCAGTTACTGCTCTGCCGCTGGAC-3′.

### RNA-seq analysis

RNA library preparation and sequencing were performed at the UCI Genomics High-throughput Facility, University of California, Irvine. Briefly, total RNA was monitored for quality control using the Agilent Bioanalyzer Nano RNA chip and Nanodrop absorbance ratios for 260/280 nm and 260/230 nm. Library construction was performed according to the Illumina TruSeq® Stranded mRNA Sample Preparation Guide. The input quantity for total RNA was 700 ng and mRNA was enriched using oligo dT magnetic beads. The enriched mRNA was chemically fragmented for 3 min. First strand synthesis used random primers and reverse transcriptase to make cDNA. After second strand synthesis the ds cDNA was cleaned using AMPure XP beads and the cDNA was end repaired and then the 3′ ends were adenylated. Illumina barcoded adapters were ligated on the ends and the adapter ligated fragments were enriched by nine cycles of PCR. The resulting libraries were validated by qPCR and sized by Agilent Bioanalyzer DNA high sensitivity chip. The concentrations for the libraries were normalized and then multiplexed together. The multiplexed libraries were sequenced on four lanes using single end 100 cycles chemistry on the HiSeq 2500. The version of HiSeq control software was HCS 2.2.58 with real time analysis software, RTA v1.18.64. Sequence alignment was performed using TopHat v2.1.1 while assembly and expression estimation was done using Cufflinks v0.12.1^79^. Reads were mapped to the mouse genome mm10 and expression values were estimated as FPKM.

DETAILS: FASTQ files were obtained from the sequencing facility and processed through the standard Tuxedo protocol^79^. Reads were then aligned to the UCSC mm10 mouse reference genome using TopHat and Bowtie2 v2.3.4. Assembled transcripts were obtained via Cufflinks with the mm10 reference annotation file. Genome assembly was obtained using Cuffmerge and expression levels (summarized to genes) were calculated using Cuffquant and then normalized via Cuffnorm to FPKM values. For each condition, 24 h time series data from six time points with three replicates each were collected. In total, expression levels of 24138 unique genes were considered for further analysis. Data was further split to pairwise time series format for comparative analysis (e.g. WT Saline vs WT Cocaine treatment, KO Saline vs KO Cocaine treatment etc).

### Bioinformatics and pathway analysis

Bioinformatics analysis was performed on RNA-seq data using JTK_CYCLE^37^ v3.1 and pipelines for CircadiOmics (circadiomics.ics.uci.edu)^80^. Pathway analysis was performed using DAVID^81^ and Reactome^82,83^ software.

Details: Statistical and bioinformatics analyses were performed based on pairwise comparisons, where the effect of cocaine treatment was analyzed while controlling the genotype or the difference between genotypes (WT or KO) were compared while controlling the treatment. Dixon’s test was performed on replicates of transcriptomic data to reduce outlier effects, filtering out up to 1 outlier replicate from each time point. For transcriptomic data, genes with consistently low expression values (FPKM < 1) were filtered out from further analysis to reduce noise. Time series data was then used to determine circadian behavior of genes using JTK_CYCLE, including the p-value for whether the time series is considered circadian, its periodicity (between 20–28 h), amplitude and phase. A gene is considered circadian if its JTK_CYCLE *p*-value passed the cutoff of 0.01. Heatmaps of circadian transcripts were generated using the R package gplots v3.0.3, where the values on each row were normalized and rows were sorted by the JTK_CYCLE phase.

The Database for Annotation, Visualization and Integrated Discovery (DAVID) pathway and Reactome analysis tools were used to identify enriched KEGG pathways for circadian genes in each condition. Pathways were ranked by the number of genes found annotated with the pathway information or with the negative natural log of p-values for enrichment, which behave similarly to z-scores where larger values indicate higher confidence.

Putative TFBS information from MotifMap^39^ were used to determine the enriched TFs in each condition. Fisher’s test was conducted comparing the relative abundance of binding sites in the promoter regions (−10000 bps to +2000 bps of transcription start site) of circadian genes in each condition, as opposed to the genomic background (defined as all 24138 genes from the RNA-seq data with FPKM > 0 at any time point). In addition, a filtering parameter of BBLS > 1, FDR < 0.25 was used to obtain high quality binding sites while TFs with motifs that are too short or degenerate (more than 50000 binding sites under the filtering criteria) were removed as they tend to be unreliable. TFs were ranked by the negative log of their Fisher p-values. Enrichment results from different pairwise comparisons were also compared in a meta-analysis to identify condition-specific TFs, in particular PPARγ which was found to be exclusively enriched in WT-Cocaine condition.

PPARγ targeted genes were filtered using a combination of MotifMap data and ChIP-Seq data from GSE64458^84^. Binding sites were searched within a smaller promoter region of −3000 bps to +1000 bps of the TSS while filtering parameters for MotifMap were kept the same as mentioned above. Other visualization and statistical analyses were performed in R or in python using pandas and scikit-learn.

### Metabolomics analysis

Metabolomic analyses were performed using p180 from the Biocrates facility (Innsbruck, Austria). Metabolite levels were measured at ZT7 after an intraperitoneal injection of saline or cocaine at a dose of 20 mg kg^−1^ at ZT3, 5 replicates each.

Statistical and bioinformatics analyses were performed based on pairwise comparisons, where the effect of cocaine treatment was analyzed while controlling the genotype or the difference between genotypes (WT or KO) were compared while controlling the treatment. Dixon’s test was performed to reduce outlier effects, filtering out up to 1 outlier replicate from each condition. Heatmaps for the metabolite profiles were generated using the RStudio software. Row z-scores are displayed and were calculated using the ‘heatmap.2’ function of the gplots package.

### Immunohistochemistry and fluorescent in situ hybridization analysis

Single immunostaining was performed on vibratome sections as described previously^85^ using anti-PPARγ antibody (1:1000; Novus Biotechnologies Cat. #NB120-19481). Nuclei staining was obtained using Draq7 (Biostatus, Cat# DR70250). For quantifications, frames of 375×375 µm/image (*n* = 4) were analyzed. ROIs were drawn around individual cells using LASX software v3.7.0 (Leica); mean gray values/cell were obtained and background subtracted. Double immunohistochemistry/in situ hybridization staining were obtained using striatal sections which were hybridized with digoxigenin (DIG)-Enkephalin or (DIG)-D1R riboprobes (RNA labeling mix; Roche, Cat# 11277073910)^29^. After incubating the probe for overnight (ON) at 60 °C, sections were washed with PBS (Phosphate Buffered Saline) 3 times (5 min), permeabilized with Triton 0.3% in PBS (15 min), blocked with normal horse serum 5% for 1 h and incubated ON with rabbit PPARγ antibody (1:1000) at 4 °C. On day 3, after 3 washes in PBS, sections were incubated for 1 h with an anti-rabbit Alexa488 (1:600, Life technologies) followed by an incubation for 1 h with anti–DIG-AP (1:5000, Roche) antibody. To amplify the signal, the HNPP (2-hydroxy-3-naphtoic acid-2’-phenylanilide phosphate) fluorescent Detection Set (Roche) was used. Quantifications were performed on confocal images (SP5, Leica) of coronal striatal slices (3 slices/animal and 3 brains/genotype/condition) using LASX v3.7.0. The number of MSNs showing the induction of PPARγ was quantified in frames of 246×246 µm/image (*n* = 3); iMSNs were defined as the number of PPARγ^+^ and Enkephalin^+^ cells while dMSNs as PPARγ^+^ and D1R + colocalizing cells.

### Chromatin immunoprecipitation

Chromatin immunoprecipitation (ChIP) procedure was performed^86^. Punches of striatum from frozen brains of two mice were pooled. Tissue was minced and double crosslinked with DSG for 20 min and 1% formaldehyde for 10 min followed by adding glycine (0.125 M final concentration) at room temperature for 10 min. After homogenizing tissue pellets in PBS, 1 ml of lysis buffer was added. Samples were sonicated (20 cycles, every cycle: 30 s ON / 30 s OFF, power high) to generate 200-500 base pairs fragments and centrifuged at 14,000 *g* at 4 °C. Supernatants were diluted in a dilution buffer (1.1% Triton X100, 1.2 mM EDTA, 16.7 mM Tris-HCl, 167 mM NaCl). The diluted chromatin was incubated with 2 mg of anti-PPARγ antibody (Abcam, Cat. # ab41928), overnight at 4 °C. To monitor the specificity of ChIP assays, samples were also immunoprecipitated with a specific-antibody isotype matched control immunoglobulin (IgG). 10ul of Dynabeads Protein G (Invitrogen, Cat. # 10003D) were added to the supernatant and incubated for 2 h at 4 °C. Beads were recovered, washed in low salt buffer, high salt buffer, LiCl buffer, followed by washing in TE for three times. Elution buffer (300 mM NaCl, 0.5% SDS, 10 mM Tris-HCl, 5 mM EDTA) was added to the washed beads, treated with RNase at 37 °C for 2 h and Proteinase K at 65 °C overnight. Equal amount of Phenol-Chloroform-Isoamyl alcohol was added to the samples and the aqueous phase was recovered. DNA was precipitated by adding 100% Ethanol, NaOAc and glycogen and kept at −20 °C overnight. Samples were centrifuged at 14,000 *g* for 30 min at 4 °C and washed with 70% ethanol followed by centrifugation at 14,000 *g* for 30 min at 4 °C. Quantitative PCRs were performed using SsoAdvanced Universal SYBR Green Supermix (Biorad, Cat. no. 172-5270), according to the manufacturer’s protocol. Primers used for ChIP analysis by RT-PCR: Kcnd1 Forward: 5′-CTCACGAGGCTAGGCAGTTC-3′, Kcnd1 Reverse: 5′-CCTTGATCGGGTGACTTGTT-3′; Gabrd Forward: 5′-CTGTTCACCTGCAATCAGGA-3′, Gabrd Reverse: 5′-GGTCTGCCCTTGAGAAATGA-3′; Adora2a Forward: 5′- AAAGATGTGGGGGAGGAGTC-3′, Adora2a Reverse: 5′-TTGCCCTTTATCGGAGCTAA-3′.

### Prostaglandins PGJ2 analysis

15-deoxy-Δ^12,14^-PGJ2 Elisa KIT (Enzo Life Sciences, Cat. no. ADI-900-023) was used to determine striatal Prostaglandin J2 concentration. ELISA tests were performed following manufacturer’s instructions. Samples were prepared as follows: striatal punches of the NAcc were minced in Phosphate buffer and the solution was then acidified by addition of HCl (2 M) to pH 3.5. Samples were centrifuged and the supernatant was passed through a C18 column (Pierce) and eluted with 20 µl of ethyl acetate. After evaporation (O/N, RT), samples were reconstituted in 250 µl of Assay Buffer and used for the Elisa assay.

### Additional statistical analyses

For all non-circadian statistics, data were analyzed either by Student’s *t*-test, or by two- or three-way ANOVA (GraphPad Prism8.3.0), followed by Tukey’s or Bonferroni’s post hoc analyses, as appropriate. Statistical significance was assigned with *p*-value < 0.05. For circadian analysis, JTK_Cycle was used with a *p*-value < 0.01 cutoff.

### Reporting Summary

Further information on research design is available in the Nature Research Reporting Summary linked to this article.

## Supplementary information

Supplementary Information

Reporting summary

## Data Availability

The GEO accession number for the RNA-seq data set reported in this paper is GSE142657. RNA-seq data was used for Figs. 2–4, and Supplementary Figs. 1–4. UCSC mm10 mouse reference genome was used for alignment. All the transcriptomic data associated with this work is publicly available on the resource circadiomics.ics.uci.edu. PPARγ ChIP-seq data used for Fig. 4 was downloaded from GEO, accession number GSE64458^84^. Source data are provided with this paper.

## Source data

Source Data
