# Supplementary material for: Cocaine-mediated circadian reprogramming in the striatum through dopamine D2R and PPARγ activation
Source: Nat Commun. 2020 Sep 7;11:4448. doi: 10.1038/s41467-020-18200-6 (PMC7477550; doi:10.1038/s41467-020-18200-6)
Supplement: Supplementary file 1 — Supplementary Information [file 41467_2020_18200_MOESM1_ESM.pdf]

Supplementary Information for Brami-Cherrier et al.

Cocaine-mediated Circadian Reprogramming in the Striatum Through Dopamine D2R and PPARγ Activation

This PDF File includes:

Supplementary Figure 1 Pathway analyses in WT and iMSN-D2RKO mice

Supplementary Figure 2 Phase and pathway analyses in WT and iMSN-D2RKO saline-treated mice

Supplementary Figure 3 Phase and pathway analyses in WT and iMSN-D2RKO cocaine-treated mice

Supplementary Figure 4 Pathway analysis of PPARγ target genes in WT cocaine-treated mice

Supplementary Table 1 Statistics for Figure 1d

Supplementary Figure 1

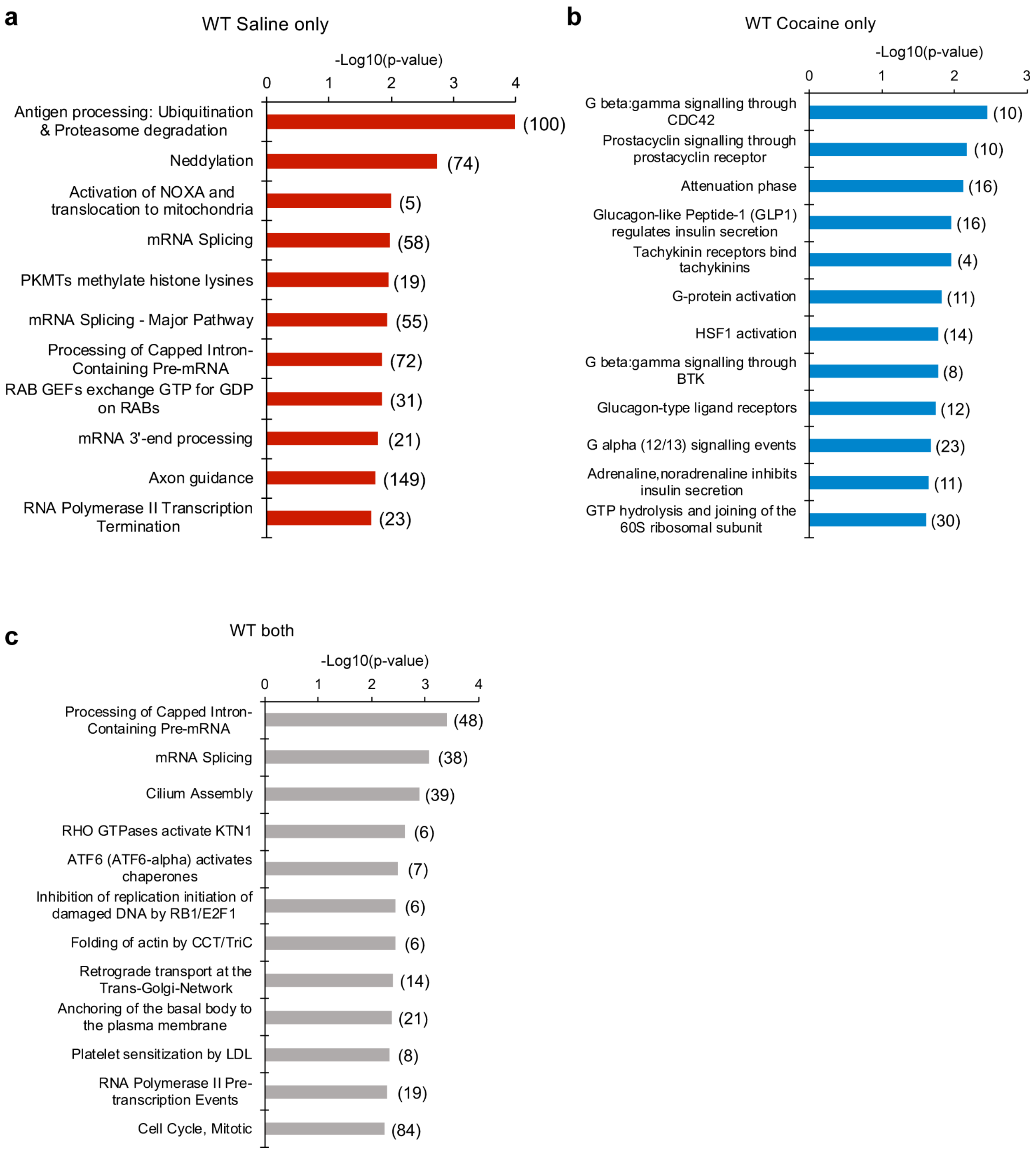

Supplementary Figure 1 Pathway analyses in WT and iMSN-D2RKO mice

a-c, Reactome Pathway analysis of circadian genes oscillating in saline only (**a**), cocaine only (**b**), and both (**c**). Bar charts represent the -Log10(p-value) of each enriched term. The number of genes identified in each pathway is shown in parenthesis.

Supplementary Figure 2

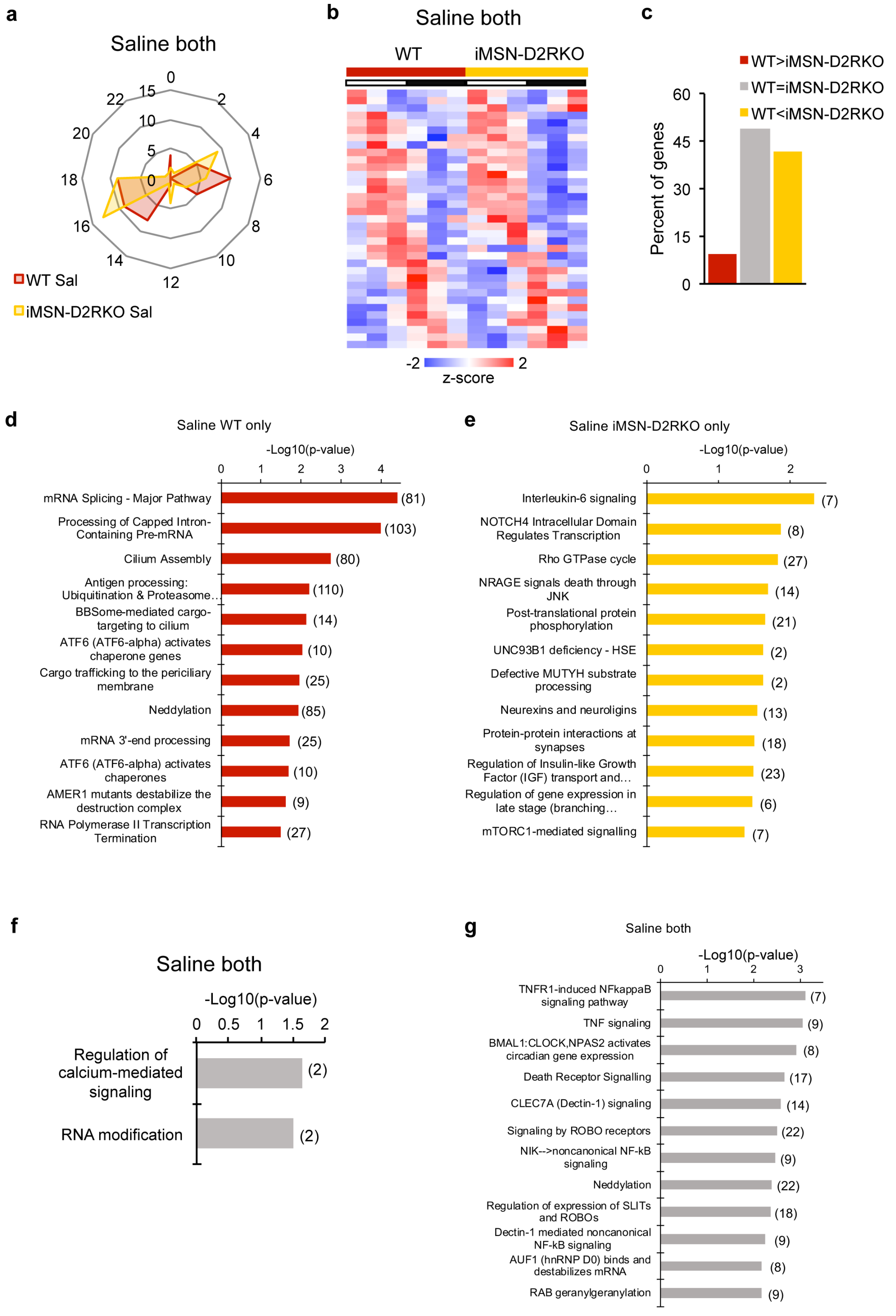

Supplementary Figure 2 Phase and pathway analyses in WT and iMSN-D2RKO saline-treated mice

a, Radar plots representing the phase analysis of genes whose expression is circadian in both WT and iMSN-D2RKO saline-treated (Sal) mice. **b**, Heat maps representing genes significantly circadian (n=3, JTK_cycle, cutoff $p<0.01$) in both WT and iMSN-D2RKO saline-treated mice. White and black bars indicate the light (ZT3, 7, 11) and dark (ZT15, 19, 23) timepoints respectively. **c**, Amplitude analysis of NAcc transcripts rhythmic in “both” WT and iMSN-D2RKO saline-treated mice. The percentage of genes with amplitude higher, lower or equal to WT is reported. **d** and **e**, Reactome Pathway analysis of circadian genes oscillating in WT only (**d**) and iMSN-D2RKO only (**e**) saline-treated mice. Bar charts represent the -Log10(p-value) of each enriched term. The number of genes identified in each pathway is shown in parenthesis. **f**, DAVID Gene Ontology Biological Process analysis of circadian genes oscillating in both WT and iMSN-D2RKO saline-treated mice. Bar charts represent the -Log10(p-value) of each enriched term. **g**, Reactome Pathway analysis of circadian genes oscillating in both WT and iMSN-D2RKO saline-treated mice. Bar charts represent the -Log10(p-value) of each enriched term. The number of genes identified in each pathway is shown in parenthesis.

Supplementary Figure 3

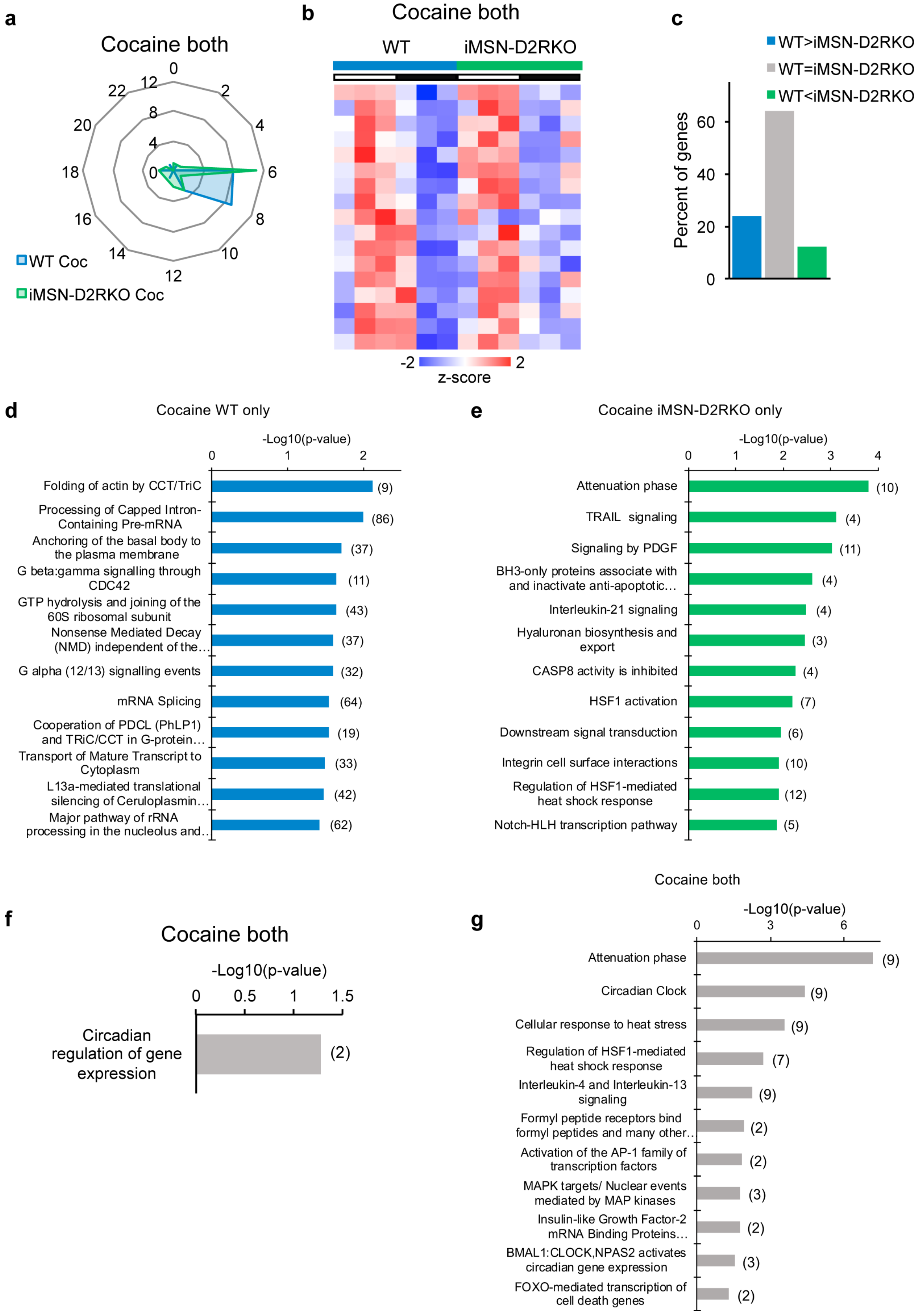

Supplementary Figure 3 Phase and pathway analyses in WT and iMSN-D2RKO cocaine-treated mice

a, Radar plots representing the phase analysis of genes whose expression is circadian in both WT and iMSN-D2RKO cocaine-treated (Coc) mice. **b**, Heat maps representing genes significantly circadian (n=3, JTK_cycle, cutoff p<0.01) in both WT and iMSN-D2RKO cocaine-treated mice. White and black bars indicate the light (ZT3, 7, 11) and dark (ZT15, 19, 23) timepoints respectively. **c**, Amplitude analysis of striatal transcripts rhythmic in "both" WT and iMSN-D2RKO cocaine-treated mice. The percentage of genes with amplitude higher, lower or equal to WT is reported. **d** and **e**, Reactome Pathway analysis of circadian genes oscillating in WT only (**d**) and iMSN-D2RKO only (**e**) cocaine-treated mice. Bar charts represent the -Log10(p-value) of each enriched term. The number of genes identified in each pathway is shown in parenthesis. **f**, DAVID Gene Ontology Biological Process analysis of circadian genes oscillating in both WT and iMSN-D2RKO cocaine-treated mice. Bar charts represent the -Log10(p-value) of each enriched term. **g**, Reactome Pathway analysis of circadian genes oscillating in both WT and iMSN-D2RKO cocaine-treated mice. Bar charts represent the -Log10(p-value) of each enriched term. The number of genes identified in each pathway is shown in parenthesis.

Supplementary Figure 4

a

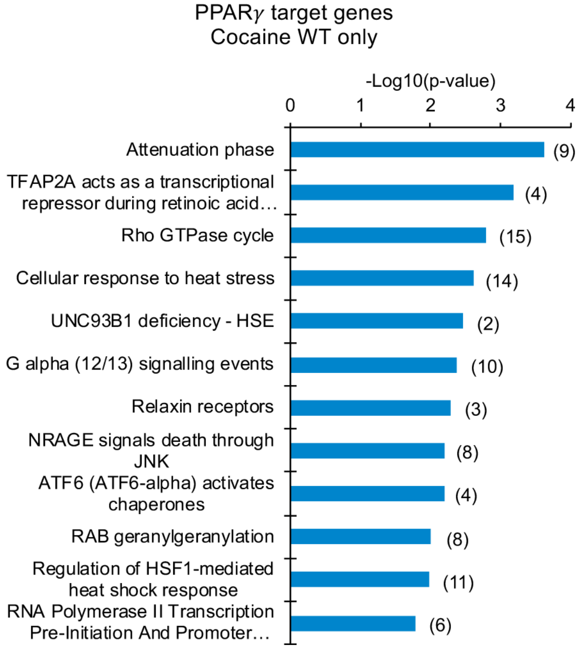

Supplementary Figure 4 Pathway analysis of PPARγ target genes in WT cocaine-treated mice

a, Reactome pathway analysis of oscillatory PPARγ target genes in cocaine-treated WT mice (n=3, JTK_Cycle, cutoff $p < 0.01$). Bar charts represent the -Log10(p-value) of each enriched term. The number of genes identified in each pathway is shown in parenthesis.

Supplementary Table 1 Statistics for Figure 1g

**Bmal1**	**SS**	**DF**	**MS**	**F (DFn, DFd)**	**P value**
Time	1.811	5	0.3622	F (5, 48) = 6.805	P<0.0001
Treatment	0.1942	1	0.1942	F (1, 48) = 3.649	P=0.0621
Genotype	0.2022	1	0.2022	F (1, 48) = 3.798	P=0.0572
Time x Treatment	0.4559	5	0.09119	F (5, 48) = 1.713	P=0.1497
Time x Genotype	0.2632	5	0.05264	F (5, 48) = 0.9889	P=0.4345
Treatment x Genotype	0.1025	1	0.1025	F (1, 48) = 1.926	P=0.1716
Time x Treatment x Genotype	0.1459	5	0.02917	F (5, 48) = 0.5481	P=0.7389
**Cry1**	SS	DF	MS	F (DFn, DFd)	P value
Time	1.743	5	0.3487	F (5, 48) = 5.429	P=0.0005
Treatment	0.006013	1	0.006013	F (1, 48) = 0.09362	P=0.7609
Genotype	0.1796	1	0.1796	F (1, 48) = 2.796	P=0.1010
Time x Treatment	0.3174	5	0.06349	F (5, 48) = 0.9885	P=0.4346
Time x Genotype	0.08598	5	0.0172	F (5, 48) = 0.2678	P=0.9285
Treatment x Genotype	0.1282	1	0.1282	F (1, 48) = 1.996	P=0.1641
Time x Treatment x Genotype	0.17	5	0.034	F (5, 48) = 0.5294	P=0.7529
**Per1**	SS	DF	MS	F (DFn, DFd)	P value
Time	0.9461	5	0.1892	F (5, 48) = 6.215	P=0.0002
Treatment	0.005365	1	0.005365	F (1, 48) = 0.1762	P=0.6765
Genotype	0.3202	1	0.3202	F (1, 48) = 10.52	P=0.0022
Time x Treatment	0.685	5	0.137	F (5, 48) = 4.500	P=0.0019
Time x Genotype	0.1803	5	0.03607	F (5, 48) = 1.185	P=0.3307
Treatment x Genotype	0.0006089	1	0.0006089	F (1, 48) = 0.02000	P=0.8881
Time x Treatment x Genotype	0.08812	5	0.01762	F (5, 48) = 0.5789	P=0.7159
**Dbp**	SS	DF	MS	F (DFn, DFd)	P value
Time	7.595	5	1.519	F (5, 48) = 6.853	P<0.0001
Treatment	0.7277	1	0.7277	F (1, 48) = 3.283	P=0.0762
Genotype	0.002965	1	0.002965	F (1, 48) = 0.01338	P=0.9084
Time x Treatment	0.9625	5	0.1925	F (5, 48) = 0.8685	P=0.5092
Time x Genotype	0.364	5	0.07281	F (5, 48) = 0.3285	P=0.8933
Treatment x Genotype	0.0834	1	0.0834	F (1, 48) = 0.3763	P=0.5425
Time x Treatment x Genotype	1.899	5	0.3799	F (5, 48) = 1.714	P=0.1495
