# Supplementary material for: Cocaine-mediated circadian reprogramming in the striatum through dopamine D2R and PPARγ activation
Source: Nat Commun. 2020 Sep 7;11:4448. doi: 10.1038/s41467-020-18200-6 (PMC7477550; doi:10.1038/s41467-020-18200-6)
Supplement: Supplementary file 2 — Reporting summary [file 41467_2020_18200_MOESM2_ESM.pdf]

natureresearch

Corresponding author(s): Paolo Sassone-Corsi; Emiliana Borrelli

Last updated by author(s): Jul 21, 2020

Reporting Summary

Nature Research wishes to improve the reproducibility of the work that we publish. This form provides structure for consistency and transparency in reporting. For further information on Nature Research policies, see Authors & Referees and the Editorial Policy Checklist .

Statistics

For all statistical analyses, confirm that the following items are present in the figure legend, table legend, main text, or Methods section.

n/a	Confirmed	
☐	☒	The exact sample size (n) for each experimental group/condition, given as a discrete number and unit of measurement
☐	☒	A statement on whether measurements were taken from distinct samples or whether the same sample was measured repeatedly
☐	☒	The statistical test(s) used AND whether they are one- or two-sided<br>*Only common tests should be described solely by name; describe more complex techniques in the Methods section.*
☐	☒	A description of all covariates tested
☐	☒	A description of any assumptions or corrections, such as tests of normality and adjustment for multiple comparisons
☐	☒	A full description of the statistical parameters including central tendency (e.g. means) or other basic estimates (e.g. regression coefficient) AND variation (e.g. standard deviation) or associated estimates of uncertainty (e.g. confidence intervals)
☐	☒	For null hypothesis testing, the test statistic (e.g. F, t, r) with confidence intervals, effect sizes, degrees of freedom and P value noted<br>*Give P values as exact values whenever suitable.*
☒	☐	For Bayesian analysis, information on the choice of priors and Markov chain Monte Carlo settings
☒	☐	For hierarchical and complex designs, identification of the appropriate level for tests and full reporting of outcomes
☒	☐	Estimates of effect sizes (e.g. Cohen's d, Pearson's r), indicating how they were calculated

Our web collection on statistics for biologists contains articles on many of the points above.

Software and code

Policy information about availability of computer code

Data collection	LASX v3.7.0 (Leica) was used to collect immunofluorescence images. Minimitter Vital View v5.0 software was used to acquire locomotor activity data. qPCR data was collected using BIO-RAD CFX Manager Software v3.1. The multiplexed libraries were sequenced using HiSeq control software version HCS 2.2.58 with real time analysis software, RTA v1.18.64.
Data analysis	R package v3.2.3, bowtie v2.3.4, TopHat v2.1.1 and Cufflinks v0.12.1 were used for raw RNA-seq analysis; JTK_CYCLE v3.1 and MotifMap (Daily et al., BMC Bioinformatics 2011) were used for RNA-seq analysis; RStudio v1.2.5033, gplots v3.0.3 package was used for heatmap generation. Software Database for Annotation, Visualization and Integrated Discovery (DAVID) v6.8 and Reactome v3.7 were used for gene ontology biological process analysis; LASX v3.7.0 (Leica) was used for immunofluorescence quantification; Clocklab software v2.72 (Matlab R2013a v9.7.0.1296695) was used for locomotor activity analyses, GraphPad Prism 8.3.0 was used for statistical analysis.

For manuscripts utilizing custom algorithms or software that are central to the research but not yet described in published literature, software must be made available to editors/reviewers. We strongly encourage code deposition in a community repository (e.g. GitHub). See the Nature Research guidelines for submitting code & software for further information.

Data

Policy information about availability of data

All manuscripts must include a data availability statement. This statement should provide the following information, where applicable:

- Accession codes, unique identifiers, or web links for publicly available datasets
- A list of figures that have associated raw data
- A description of any restrictions on data availability

RNA-seq data has been deposited in the Gene Expression Omnibus (GEO) with the accession number GSE142657. RNA-seq data was used for Figure 2, Figure 3, Figure 4, and Supplementary Figures 1-4. UCSC mm10 mouse reference genome was used for RNA-seq alignment. All the transcriptomic data associated with this work is publicly available on the resource circadiomics.ics.uci.edu. PPARg ChIP-seq data was downloaded from GEO accession number GSE64458 was used for Figure 4.

Field-specific reporting

Please select the one below that is the best fit for your research. If you are not sure, read the appropriate sections before making your selection.

[x] Life sciences [ ] Behavioural & social sciences [ ] Ecological, evolutionary & environmental sciences

For a reference copy of the document with all sections, see nature.com/documents/nr-reporting-summary-flat.pdf

Life sciences study design

All studies must disclose on these points even when the disclosure is negative.

Sample size	Sample size was chosen based on numbers we used for previous publications (Lewis et al., Cell Rep 2020, Cervantes et al., iScience 2020) reporting similar experiments with a minimum of n=3.
Data exclusions	No data were excluded.
Replication	Each experiment was repeated at least twice to ensure the reproducibility of the results.
Randomization	Animals were randomly assigned to a treatment group and a timepoint for sacrifice.
Blinding	Investigators were blinded for group allocation, data collection and analysis.

Reporting for specific materials, systems and methods

We require information from authors about some types of materials, experimental systems and methods used in many studies. Here, indicate whether each material, system or method listed is relevant to your study. If you are not sure if a list item applies to your research, read the appropriate section before selecting a response.

Materials & experimental systems

n/a	Involved in the study	
[ ]	[x]	Antibodies
[x]	[ ]	Eukaryotic cell lines
[x]	[ ]	Palaeontology
[ ]	[x]	Animals and other organisms
[x]	[ ]	Human research participants
[x]	[ ]	Clinical data

Methods

n/a	Involved in the study	
[x]	[ ]	ChIP-seq
[x]	[ ]	Flow cytometry
[x]	[ ]	MRI-based neuroimaging

Antibodies

Antibodies used	anti-PPARg antibody (1:1000 for IF, Novus Biotechnologies Cat.# NBP1-61399; 2 mg for ChIP, abcam Cat.# ab41928), anti-rabbit Alexa488 (1:600 for IF, Life 481 technologies Cat.# A-11034), anti–DIG-AP (1:5000 for in situ, Roche Cat.# 11093274910)
Validation	PPARg antibody Novus Biotechnologies Cat.# NBP1-61399 was validated per manufacture's website for IF in mouse brain tissue; Abcam Cat.# ab41928 was validated for ChIP in mouse by manufacture, labeled as "ChIP-grade" and previously reported in Murakami et al. EMBO Rep 2016.

Animals and other organisms

Policy information about studies involving animals; ARRIVE guidelines recommended for reporting animal research

Laboratory animals	WT and iMSN-D2RKO male mice (C57BL/6J background) were treated and sacrificed at 3-5 months old. Animals were individually housed for locomotor analysis, group housed otherwise, fed ad. libitum in 12h light/dark cycle, ~25ºC, 45-60% humidity.
Wild animals	This study did not involve wild animals.
Field-collected samples	This study did not involve samples collected from the field.
Ethics oversight	All animal experimental procedures were approved by the Institutional Animal Care and Use Committee of the University of California, Irvine.

Note that full information on the approval of the study protocol must also be provided in the manuscript.
